# A Clinical Perspective of the Multifaceted Mechanism of Metformin in Diabetes, Infections, Cognitive Dysfunction, and Cancer

**DOI:** 10.3390/ph15040442

**Published:** 2022-04-02

**Authors:** Elaine Chow, Aimin Yang, Colin H. L. Chung, Juliana C. N. Chan

**Affiliations:** 1 Department of Medicine and Therapeutics, The Chinese University of Hong Kong, Prince of Wales Hospital, Hong Kong 999077, China; e.chow@cuhk.edu.hk (E.C.); aiminyang@cuhk.edu.hk (A.Y.); 1155125450@link.cuhk.edu.hk (C.H.L.C.); 2 The Hong Kong Institute of Diabetes and Obesity, The Chinese University of Hong Kong, Prince of Wales Hospital, Hong Kong 999077, China; 3 Phase 1 Clinical Trial Centre, The Chinese University of Hong Kong, Prince of Wales Hospital, Hong Kong 999077, China

**Keywords:** metformin, diabetes, mechanisms, anticancer action, infections, cognition, cardioprotection

## Abstract

In type 2 diabetes, ecological and lifecourse factors may interact with the host microbiota to influence expression of his/her genomes causing perturbation of interconnecting biological pathways with diverse clinical course. Metformin is a plant-based or plant-derived medicinal product used for the treatment of type 2 diabetes for over 60 years and is an essential drug listed by the World Health Organization. By reducing mitochondrial oxidative phosphorylation and adenosine triphosphate (ATP) production, metformin increased AMP (adenosine monophosphate)-activated protein kinase (AMPK) activity and altered cellular redox state with reduced glucagon activity, endogenous glucose production, lipogenesis, and protein synthesis. Metformin modulated immune response by directly reducing neutrophil to lymphocyte ratio and improving the phagocytic function of immune cells. By increasing the relative abundance of mucin-producing and short-chain-fatty-acid-producing gut microbes, metformin further improved the host inflammatory and metabolic milieu. Experimentally, metformin promoted apoptosis and reduced proliferation of cancer cells by reducing their oxygen consumption and modulating the microenvironment. Both clinical and mechanistic studies support the pluripotent effects of metformin on reducing cardiovascular–renal events, infection, cancer, cognitive dysfunction, and all-cause death in type 2 diabetes, making this low-cost medication a fundamental therapy for individualization of other glucose-lowering drugs in type 2 diabetes. Further research into the effects of metformin on cognitive function, infection and cancer, especially in people without diabetes, will provide new insights into the therapeutic value of metformin in our pursuit of prevention and treatment of ageing-related as well as acute and chronic diseases beyond diabetes.

## 1. Introdaction

In 2021, an estimated 537 million people or 10.5% of the world’s population were affected by diabetes, the majority having type 2 diabetes (T2D) with major healthcare and socioeconomic implications [1]. Pharmacological treatment plays an important role in the prevention and treatment of T2D. Understanding the physiology of glucose homeostasis as elegantly defined by Gerich JE is critical to understanding the mechanisms of these glucose-lowering drugs (GLDs) [2]. Glucose and free fatty acids are the main energy substrates essential for survival with excess energy stored as glycogen in the liver and muscle, and triglycerides in adipose tissues. Under metabolic stress with low oxygen and glucose supply, lactate and ketones are alternative fuels. Chronic exposure to high glucose can lead to glucotoxicity causing dysregulation of metabolic, vascular, inflammatory, and cell signaling pathways resulting in widespread organ damage [3].

For survival purpose, the human body possesses a set of mechanisms to maintain blood glucose within a narrow range of 4–8 mmol/L, irrespective of energy intake or expenditure. Type 2 diabetes is characterized by chronic hyperglycemia due to non-suppression of glucagon and reduced post-prandial insulin secretion, often worsened by obesity-associated insulin resistance. During fasting, glycogen and triglyceride are broken down to release glucose and free fatty acids, which are interchangeable through the Randle cycle as energy source. Glucose can also be generated through gluconeogenesis where protein can be broken down by counter-regulatory hormones, such as cortisol, growth hormone, and catecholamines into amino acids which are then converted to glucose to maintain energy balance. In a prandial state, insulin is released to promote glycogen and fat storage while excess glucose is excreted through the kidney, albeit with reabsorption. To date, all GLDs utilize some of these mechanisms to regulate blood glucose levels by reducing energy intake, suppressing endogenous glucose production, reducing glucose reabsorption from gut or kidney, and/or redistributing energy storage [4].

## 2. Metformin Pharmacology and Mechanisms of Action

### 2.1. Metformin Pharmacology

Metformin is currently recommended as the first-line GLD in patients with T2D [5]. This plant-derived medicinal product has been used in the treatment of T2D for over 60 years [6]. Galegine or guanidine is a chemical extracted from the herbal plant, *Galega officinalis* [7]. Metformin is a synthetic guanidine with two coupled molecules (biguanide) and additional chemical substitutions. Metformin is transported into the cell via organic transporter-3 (OCT-3) and OCT-1. It is mainly absorbed from the upper small intestine with an absolute bioavailability of 50–60%. The half-life of plasma level of metformin ranges from 0.9–2.6 h although the latter may vary with different formulations with reports of prolonged half-life due to accumulation in other tissues such as red blood cells [8]. Metformin is excreted unchanged in the urine. Using C^11^ positron emission tomography, orally administered metformin is mainly concentrated in the liver, kidneys, and bladder with the highest concentrations detected in the liver [9] and jejunal sites [10].

Apart from its low oral bioavailability and short half-life, gastro-intestinal side effects are not uncommon with metformin therapy. Drug delivery systems have been designed to overcome these limitations associated with conventional dosage forms of metformin. Development of novel formulation (e.g., microparticles, and nanoparticles) may improve its bioavailability, reduce the dosing frequency and decrease gastrointestinal side effects with improved effectiveness in the treatment of diabetes and, possibly, cancer [11].

In the last decade or so, there have been a large number of publications on the clinical effects and molecular mechanisms of metformin, with the latter being elegantly summarized in the latest review by LaMoia et al. [12]. In the present article, we aim to interpret these molecular studies in the lens of practicing physicians and highlight the knowledge gap in translating this evidence to clinical practice especially in areas of unmet needs, such as cancer, infection, and cognitive dysfunction in people with or without diabetes.

### 2.2. Inhibition of Mitochondrial Metabolism and Endogenous Glucose Production

**Figure 1:**
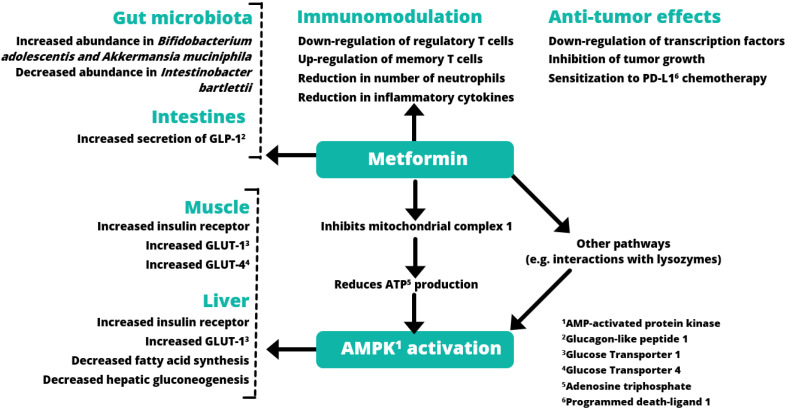
Mechanisms of metformin. The multifaceted nature of the mechanisms of metformin targeting different organs, including liver, muscle, and gastrointestinal tract, including the microbiota, results in glucose-lowering, anti-inflammatory, and anti-cancer effects through AMPK and non-AMPK dependent pathways (adapted from references [16,17,18,19]).

The primary action of metformin is mediated through its effects on mitochondrial metabolism. Metformin with positive charges tends to accumulate within the mitochondria due to the action potentials across its inner membranes. Within the mitochondria, metformin inhibits Complex I of the electron transport chain leading to reduced oxidative phosphorylation with decreased amount of adenosine triphosphate (ATP) as an energy unit. The increased adenosine monophosphate (AMP) to ATP ratio activates the AMP-activated protein kinase (AMPK) with reduced energy storage. The cellular increase in AMP inhibits adenylate cyclase activity with reduced glucagon signaling needed for glycogenolysis to release glucose [13]. Metformin also reduced mitochondrial glycerophosphate dehydrogenase (mGPD). This leads to an altered cellular redox state with reduced endogenous glucose production including reduced conversion of lactate and glycerol to glucose and hepatic gluconeogenesis [14] (Figure 1). At the same time, AMPK activation increases the activity of insulin receptors and translocation of glucose transporters (GLUT) including ubiquitously expressed GLUT-1 and GLUT-4 expressed in muscle to promote glucose uptake in the peripheral tissues [15].

Due to its inhibition of mitochondrial respiratory chain complex 1 which favours anaerobic respiration, metformin may increase accumulation of lactic acid with increased risk of lactic acidosis. However, metformin-associated lactic acidosis usually occurs within a setting of increased production due to hypoxia with acute cardiopulmonary events or sepsis and renal dysfunction with reduced clearance. In a non-stress situation, the production of lactate by metformin may improve the efficiency of energy metabolism. After donating its proton, lactic acid becomes lactate which is a more efficient cell-to-cell shuttle for delivery of oxidative and gluconeogenic substrates. Through direct uptake and oxidation of lactate produced elsewhere, metabolically active organs (such as cardiomyocytes, liver, and renal cells) can utilize lactate for immediate use without relying on glycolysis and endogenous glucose production [20].

Among metformin users, the incidence of lactic acidosis had been estimated to range from 2 to 9 cases per 100,000 person-years [21] with most of these events occurring within a setting of multi-organ dysfunction. A large body of real-world evidence supported the safety and efficacy of metformin with increasing prescription during the past two decades [22,23,24]. Apart from its benefits in glucose metabolism, there is a growing body of evidence from clinical trials and observational studies indicating that metformin might prevent or alleviate complications and co-morbidities of T2D such as cardiovascular diseases (CVD), chronic kidney disease (CKD), obesity, cancer, and infections including pneumonia, tuberculosis, and, more recently, coronavirus disease (COVID-19) [25], mediated by both AMPK-dependent and AMPK-independent mechanisms [26].

### 2.3. Metformin and Hepatic Gluconeogenesis

There are different mechanisms through which metformin can regulate hepatic gluconeogenesis. In terms of transcription alteration, metformin-activated AMPK directly downregulates expression of gluconeogenic genes. The accumulation of AMP inhibits adenylate clyclase and reduces cyclic AMP (cAMP) level which prevents transcription of gluconeogenic genes mediated by the cAMP-response element-binding protein (CREB). [12]. Besides, metformin inhibits mitochondrial glycerol-3-phosphate dehydrogenase-2 (GPD2) which converts glycerol to dihydroxyacetone phosphate [12]. By inhibiting GPD2, metformin increases glycerol and glycerol 3-phosphate levels with reduced gluconeogenesis. GPD2 inhibition is a redox-dependent enzyme and is part of the α-glycerophosphate shuttle. The latter maintains the NADH/NAD+ (nicotinamide adenine dinucleotide) ratio. By inhibiting GPD2, metformin alters the NADH/NAD+ ratio and cytosol redox state and inhibits gluconeogenesis from lactate and glycerol [12].

### 2.4. Modulation of Gut Microbiota and Inflammation

Metformin efficacy and tolerance are closely linked with the gastrointestinal physiology with accumulating evidence supporting the role of gut microbiota in glucose metabolism [27]. There are approximately 100 trillion micro-organisms, including bacteria, viruses, fungi, and protozoa, in the gastrointestinal tract of a typical person. While the human genome consists of about 23,000 genes, the collective genomes of this microbiota encode over three million genes producing thousands of metabolites which can influence the health and phenotypes of the host. Both animal and clinical studies indicated that different abundance of microbiota was associated with changes in inflammatory microenvironment and production of bile acids and short-chain fatty acids (SCFA) which could contribute to onset and progression in T2D [28].

In clinical trials, metformin-treated patients showed increased abundance of beneficial bacteria such as *Akkermansia muciniphila* which was negatively associated with the risk of T2D. In a 4-month, double-blind, placebo-controlled study involving treatment-naive patients with T2D [28], metformin treatment increased *Akkermansia muciniphila*, *Bifidobacterium adolescentis* and *Lactobacilius fermentium*, and decreased *Intesinibacter bartlettii* and *Clostridium* spp. Amongst these species, changes in *Bifidobacterium adolescentis* were directly related to the dosage of metformin. In this short-term study, there was no difference in body weight, body fat, or fasting plasma insulin between placebo and metformin although glycated haemoglobin (HbA1c) and fasting plasma glucose were reduced in the metformin group [29]. Two other clinical trials reported similar findings where metformin treatment increased mucin-producing *Akkermansia muciniphila*, and SCFA-producing microbes [30] including *Butyrivibrio*, *Bifidobacterium bifidum*, *Megasphaera*, and *Prevotella* [31]. These microbes utilized different dietary substrates to produce an array of metabolites which can influence the host microenvironment with beneficial metabolic effects. Whilst *Bifidobacterium* species had been shown to induce gene expression involved in carbohydrate metabolism [32], *Prevotella* species could degrade starch [31] and metabolize fructose to produce medium-chain carboxylic acids with improved fuel transport [33]. In a study involving newly diagnosed patients with T2D, 3-day treatment with metformin reduced the genus *Bacteroides fragilis* and increased the bile acid, glycoursodeoxycholic acid, which might contribute to the anti-inflammatory and metabolic effects of metformin [34]. Intriguingly, metformin had been shown to lengthen lifespan of the nematode worm, *Caenorhabditis elegans*, via changes in microbial folate and methionine metabolism [35].

## 3. Clinical Evidence for Pleiotropic Effects of Metformin

**Figure 2:**
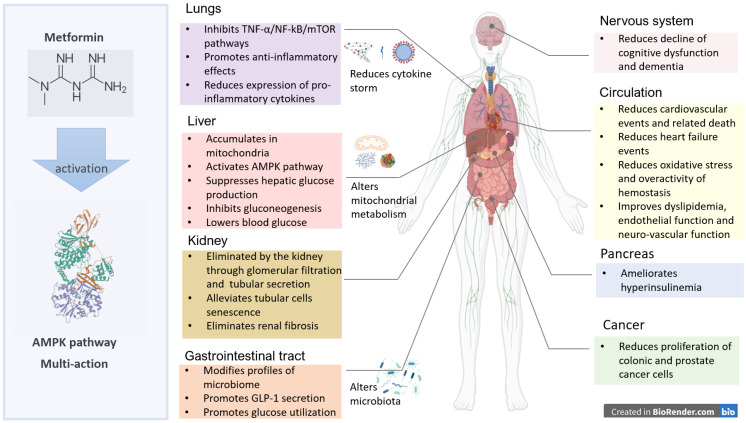
Clinical benefits of metformin in multiple systems. The multi-targeted actions of metformin are mediated both by the adenosine monophosphate activated protein kinase (AMPK) pathway and non-AMPK pathways. In the liver, metformin reduces glycogenolysis, hepatic glucose production, and gluconeogenesis [37]. In the lung, metformin modulates the tumor necrosis factor (TNF)-α/NF-kB/mammalian target of rapamycin (mTOR) pathways and expression of pro-inflammatory cytokines. In the intestines, metformin modifies gut microbiome and promotes incretin (e.g., glucagon-like peptide 1, GLP-1) secretion with increased glucose utilization. In the nervous system, metformin reduces amyloid plaque formation and decline of cognitive function. In the circulatory systems, metformin improves dyslipidemia and endothelial dysfunction with reduced cardiovascular–renal events. Metformin reduces site-specific cancer events, including prostate and liver, in part due to amelioration of insulin resistance with reduced activation of insulin/insulin-like growth factor (IGF-1). Metformin is eliminated by the kidney. Metformin alleviates podocyte loss, mesangial cells apoptosis, and tubular cells senescence through AMPK-mediated signaling pathways. In chronic kidney disease, renal fibrosis is ameliorated by metformin, mainly via AMPK activation. Reduced glomerular filtration and tubular secretion may lead to accumulation of metformin and increased risk of lactic acidosis, especially in stress situations [17] (adapted from reference [13]).

The multi-targeted actions of metformin are mediated both by the AMPK pathway ubiquitous in all cells for energy metabolism and non-AMPK mechanisms [36]. The clinical benefits of metformin have been reported in liver, pancreas, lungs, and gastrointestinal tract as well as cardiovascular–renal and nervous systems (Figure 2).

**Figure 3:**
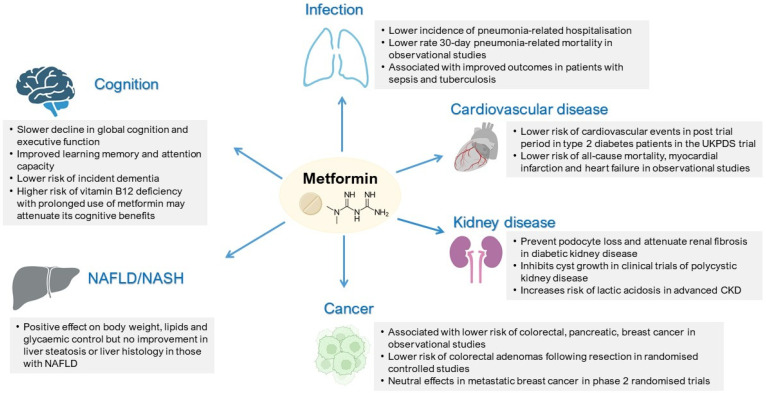
Summary of clinical effects of metformin in different disease conditions. NAFLD, Non-alcoholic fatty liver disease (NAFLD); NASH, non-alcoholic steatohepatitis; CKD, chronic kidney disease; and UKPDS, United Kingdom Prospective Diabetes Study.

In this section, we reviewed the potential molecular mechanisms and clinical evidence regarding the effects of metformin in closely related conditions, including cardiovascular–renal disease, infection, cancer, non-alcoholic fatty liver disease (NAFLD), and cognitive dysfunction. Figure 3 summarizes the clinical effects of metformin in different disease conditions.

### 3.1. Putative Mechanisms of Metformin on Cardiovascular Systems

Diabetes is a major risk factor for CVD and CKD. In the 10-year follow-up analysis of the United Kingdom Prospective Diabetes Study (UKPDS), treatment with metformin was associated with reduced cardiovascular events and all-cause mortality [38]. The Diabetes Prevention Program conducted in the United States of America (USA) was the largest and longest clinical trial assessing the effects of metformin in people with impaired glucose tolerance (IGT). In this study, metformin was confirmed to prevent T2D which was translated to reduction in cardiovascular events in the post-trial period [39]. Apart from these two large randomised controlled trials (RCTs), most of the evidence in support of the cardiovascular–renal effects of metformin were inferred from systematic reviews and meta-analysis of observational studies (Table 1).

In a meta-analysis of RCTs including 2079 patients with T2D [40], metformin use was associated with reduced risk of cardiovascular death, myocardial infarction, and peripheral vascular disease compared with non-use of metformin. The most consistent benefits were observed for all-cause mortality with up to 16% risk reduction, albeit with an increased risk of stroke by 48% [39]. In a more recent meta-analysis involving 1,160,254 patients with T2D, metformin use was associated with decreased cardiovascular mortality (relative risk, RR = 0.44 (95% CI: 0.34–0.57) and incidence of CVD (RR = 0.73, 95% CI: 0.59–0.90)) [41]. However, the risk association of metformin with myocardial infarction and heart failure amongst patients with T2D [42,43] was not always consistent [44], with many of these meta-analyses and systematic reviews having low or critically low quality [25,44,45]. The heterogeneous clinical profiles, study design, and settings contributed towards these controversies calling for more RCTs with better-defined settings, populations, and study design, preferably with comparative drugs [44].

**Table 1:** Studies on the association of metformin use with clinical events in patients with type 2 diabetes.

Author/ Year	Study Design	Region	No. of Participants	No. of Cases	Follow-Up (Years)	Comparations and Outcomes	Main Conclusion
Raee, 2017 [46]	Cohort	Iran	717	446	3.0	Glyburide versus metformin All-cause mortality: HR = 0.27, 95% CI: 0.10–0.73 Cardiovascular mortality: HR = 0.12, 95% CI: 0.20–0.66	Compared with metformin, glyburide was associated with increased all-cause and cardiovascular mortality in patients with diabetes.
Scheller, 2014 [47]	Retrospective cohort	Denmark	84,756	83,528	5.0	Sitagliptin versus metformin All-cause mortality: HR = 1.25, 95% CI: 0.92–1.71 Incidence of CVD: HR = 1.22, 95% CI: 0.96–1.61	Compared with metformin monotherapy, sitagliptin monotherapy was not associated with increased risk of all-cause mortality or CVD.
Roumie, 2012 [48]	Retrospective cohort	USA	253,690	155,025	5.5	Sulfonylurea versus metformin CVD (acute myocardial infarction and stroke) or death: HR = 1.21, 95% CI: 1.13–1.30	Compared with metformin, use of sulfonylureas was associated with an increased hazard of CVD events or death.
Roumie, 2017 [49]	Retrospective cohort	USA	131,972	65,986	0.9–1.1	Sulfonylurea versus metformin Heart failure and cardiovascular death: HR = 1.32, 95% CI: 1.21–1.43	Compared with metformin, sulfonylurea had a higher risk of heart failure and cardiovascular death.
Johnson, 2002 [50]	Cross-sectional	Canada	4183	1150	5.1	Metformin versus sulfonylurea All-cause mortality: OR = 0.60, 95% CI: 0.49–0.74 cardiovascular–related mortality: OR = 0.53, 95% CI: 0.41–0.68	Metformin therapy, alone or in combination with sulfonylurea, was associated with reduced all-cause and cardiovascular mortality.
Ekstrom, 2012 [51]	Register-based cohort	Sweden	32,152	14,696	3.9	Other-GLDs versus metformin Incidence CVD: HR = 1.02, 95% CI: 0.93–1.12 All–cause mortality: HR = 1.13, 95% CI: 1.01–1.27	Metformin showed lower risk than insulin for CVD and all-cause mortality and slightly lowered risk for all-cause mortality compared with other GLDs.
Pantalone, 2012 [52]	Retrospective cohort	USA	23,915	12,774	2.2	Glipizide, glyburide, glimepiride versus metformin All-cause mortality: - glipizide: HR = 1.64 (1.39–1.94) - glyburide: HR = 1.59 (1.35–1.88) - glimepiride: HR = 1.68(1.37–2.06)	Glipizide, glyburide and glimepiride were associated with an increased risk of overall mortality versus metformin.
Charytan, 2019 [53]	Clinical trails	USA	4038	591	4.0	Metformin versus non-metformin All-cause mortality: HR = 0.49, 95% CI: 0.36–0.69 Cardiovascular death: HR = 0.49, 95% CI: 0.32–0.74 Cardiovascular composite: HR = 0.67, 95% CI: 0.51–0.88 Kidney disease composite: HR = 0.77, 95% CI: 0.61–0.98 ESKD (end stage kidney disease): HR = 1.01,95% CI: 0.65–1.55	Metformin might be safer for use in CKD than previously considered with reduced risk of death and cardiovascular events in individuals with stage 3 CKD.
Cheng, 2014 [54]	Retrospective cohort	Taiwan	14,856	10,857	4.0	Metformin versus non-metformin Incidence stroke: HR = 0.38, 95% CI: 0.35–0.42	Compared with non-metformin use, metformin use was associated with lower risk of stroke especially in high-risk patients
Mogensen, 2015 [55]	Retrospective cohort	Danish	28,236	16,910	13.0	Sulfonylureas + metformin versus metformin/metformin + insulin All-cause mortality: RR = 1.81, 95% CI: 1.63–2.01 cardiovascular death: RR = 1.35, 95% CI: 1.14–1.60 Composite endpoint (myocardial infarction, stroke and cardiovascular death): RR = 1.25, 95% CI: 1.09–1.42	In combination with insulin, the use of sulfonylureas was associated with increased mortality compared with metformin.
Evans, 2006 [56]	Retrospective cohort	UK	5617	2286	8.0	Sulfonylurea versus metformin All-cause mortality: HR = 1.43, 95% CI: 1.15–1.77 Cardiovascular mortality: HR = 1.70, 95% CI: 1.18–2.45	Patients treated with sulfonylureas only, or combinations of sulfonylureas and metformin, were at higher risk of adverse cardiovascular outcomes than those treated with metformin alone.
Sillars, 2010 [57]	Retrospective cohort	Australia	1271	390	10.4	Metformin–sulphonylurea versus diet and metformin monotherapy All-cause mortality: HR = 0.82, 95% CI: 0.58–1.23 Cardiovascular mortality: HR = 0.82, 95% CI: 0.53–1.27	Combination metformin–sulphonylurea appeared to be as safe as other blood glucose-lowering therapies used in type 2 diabetes.
Morgan, 2014 [58]	Retrospective cohort	UK	80,999	68,139	2.9–3.1	Sulfonylurea versus metformin All-cause mortality: HR = 1.27, 95% CI: 1.02–1.58 MACE (adverse cardiovascular events): HR = 0.81, 95% CI: 0.57–1.15	All-cause mortality was increased in patients prescribed with sulphonylureas compared with metformin monotherapy.
Breunig, 2014 [59]	Retrospective cohort	USA	6271	5548	1.6	Rosiglitazone, pioglitazone versus metformin Incidence of heart failure: Rosiglitazone: HR = 1.57, 95% CI: 1.15–2.15	Compared with metformin, there appeared to be higher risk of heart failure in patients started on rosiglitazone but not pioglitazone
Fung, 2015 [60]	Retrospective cohort	Hong Kong	11,293	7493	5.0	Metformin versus non–metformin All-cause mortality: HR = 0.73, 95% CI: 0.58–0.90 Incidence CVD: HR = 0.72, 95% CI: 0.60–0.87 Incidence of coronary heart disease: HR = 0.67, 95% CI: 0.52–0.86 Incidence of stroke: HR = 0.75, 95% CI: 0.57–0.98 Incidence of CKD (eGFR < 30): HR = 1.08, 95% CI: 0.84–1.38	Patients who were started on metformin monotherapy showed improvement in many of the clinical parameters and a reduction in all-cause mortality and CVD events than lifestyle modifications alone

Note: CVD, cardiovascular disease; HR, hazard ratio; CI, confidence interval; RR, relative risk; GLDs, glucose-lowering drugs; CKD, chronic kidney disease; and eGFR, estimated glomerular filtration rate.

#### 3.1.1. Metformin and Endothelial Dysfunction, Inflammation and Oxidative Stress

Pending definitive evidence on clinical outcomes, metformin has been shown to improve surrogate markers of CVD, incuding endothelial dysfunction, dyslipidemia, and systemic inflammation [38,61,62]. Metformin improves endothelial dysfunction by increasing nitric oxide synthase (eNOS) with increased generation of nitric oxide (NO), a potent vasodilator. Other mechanisms include suppression of mitochondrial complex 1, stimulation of AMPK and inhibition of apoptosis [63]. In experimental studies, AMPK activation by therapeutically relevant concentrations of metformin (50–500 μM) increased NO via increasing eNOS phosphorylation and eNOS interaction with heat shock protein 90 (HSP90) [64]. Metformin restores the impaired eNOS-HSP90 interaction in high-glucose exposed endothelial cells [64]. The eNOS activating effect of metformin (250 mg/kg/d) has also been demonstrated in endothelial progenitor cells from streptozotocin (STZ)-induced diabetic mice [65]. Metformin further attenuates glucose-induced endothelial dysfunction through enhancing guanosine 5′triphosphate cyclohydrolase 1 (GTPCH1) mediated eNOS recoupling and NADPH oxidase inhibition [66]. Metformin raises the tissue concentration of hydrogen sulfide (H2S), which is a major endothelium-derived hyperpolarising factor (EDHF) that causes vascular endothelial and smooth muscle cell hyperpolarization and vasorelaxation by activating the ATP-sensitive potassium channels through cysteine S-sulfhydration [67].

Metformin may exert anti-inflammatory effects and reduce oxidative stress via multiple pathways. This can occur via an AMPK-dependent inhibition of the inhibitory-kB kinase (IKK)/IkBalpha/NF-kB [67]. Metformin also inhibits tumor necrosis factor (TNF)-α induced gene expression of cell adhesion molecules that contribute to monocyte adhesion which promotes atherogenesis. Acting via AMPK, metformin also exhibits epigenetic effects and phosphorylates multiple substrates, including histone acetyltransferases class II histone deacetylases (HDACs) and DNA/histone methyltransferase [63]. For example, metformin may increase Sirtuin 1 (SIRT1) activity and protect against hyperglycemia-induced metabolic memory resulting in endothelial dysfunction [68].

#### 3.1.2. Metformin on Blood Flow and Haemostasis

Several studies have shown favourable effects of metformin on blood flow. Metformin increased haemodynamic responses to L-arginine, the precursor of vasodilatory NO [69]. Metformin also lowered levels of asymmetric dimethylarginine, an endogenous inhibitor of nitric oxide synthase (NOS) in T2D. Metformin reduced platelet activity and haemostasis with reduced clot formation [70]. In clinical studies, metformin reduced plasminogen activator inhibitor-1 (PAI-1) with increased fibrinolysis [71], although results are not always consistent [70]. In in vitro studies, metformin had been shown to reduce platelet activation by reducing extracellular mitochondrial DNA (mtDNA) release [72].

#### 3.1.3. Metformin and Kidney Disease

The occurrence of CKD in patients with T2D markedly amplified the risk of CVD [73,74]. In patients with mild to moderate CKD (stages 3a and 3b with respective estimated glomerular filtration rate [eGFR] 45–59 and 30–44 mL/min/1.73 m^2^), the incidence of death due to CVD was considerably higher than that due to kidney failure, with these patients having the double burden of CVD and end-stage kidney disease (ESKD) [75]. Metformin reduces blood glucose without causing weight gain and hypoglycemia [76] which are conducive to the prevention of CVD. This low risk of hypoglycemia is particularly relevant to patients with CKD who are at high risk for both CVD and hypoglycemia which are closely associated. Despite the lack of definitive evidence from RCTs, cohort analyses, and real-world evidence suggested neutral or beneficial cardiovascular–renal effects of metformin in patients with T2D at different stages of CKD (Table 2). These clinical findings are supported by experimental findings where metformin use in rats with CKD prevented progression of renal dysfunction, reduced vascular calcification, and inhibited high bone turnover with reduced renal expression of cellular infiltration, fibrosis, and inflammation [77].

**Table 2:** Cohort studies on the association of metformin use with cardiovascular–renal outcomes at different CKD stages.

Author/ Year	Study Design	Sample Size	Comparation	Duration/ Dose	Outcomes, Hazard Ratio (95% CI)	Main Conclusion
Whitlock, 2020 [78]	Retrospective Cohort (2006–2017) FU: 1.4 vs. 1.1 years	21,996 (metformin: 19,990)	metformin vs. sulfonylurea among patients with T2D (age > 18 years)	NA	All-cause mortality: Overall: 0.48 (0.40–0.58) eGFR ≥90: 0.38 (0.27–0.53) eGFR 60–89: 0.42 (0.31–0.56) eGFR 45–59: 0.92 (0.53–1.61) eGFR 30–44: 0.85 (0.46–1.57) eGFR <30: 1.51 (0.58–3.95) CVD: Overall: 0.64 (0.41–1.00) eGFR ≥90: 0.78 (0.52–1.2) eGFR 60–89: 0.86 (0.45–1.64) eGFR 45–59: 0.62 (0.3–1.29) eGFR 30–44: 0.85 (0.46–1.57) eGFR <30: 0.56 (0.18–1.69)	Metformin use was associated with lower risk for all-cause mortality, cardiovascular events, and major hypoglycemic episodes when compared with sulfonylureas. CKD was a significant effect modifier for all-cause mortality, but not for cardiovascular events or major hypoglycemic episodes.
Kwon, 2020 [79]	Retrospective Cohort (2001–2016) FU: 7.3 years	10,426	metformin vs. non-metformin among patients with type 2 diabetes kidney disease	Duration and dose	All-cause mortality: Overall: 0.48 (0.40–0.58) eGFR ≥45: 0.38 (0.27–0.53) eGFR 45–30: 0.42 (0.31–0.56) eGFR <30: 0.55 (0.37–0.81) ESKD: Overall: 0.67 (0.58–0.77) eGFR ≥45: 0.62 (0.51–0.76) eGFR 45–30: 0.73 (0.54–0.99) eGFR <30: 0.87 (0.67–1.12)	Metformin usage in advanced CKD patients, especially those with CKD 3b, was associated with reduced risk of all-cause mortality and incident ESKD. Metformin did not increase the risk of lactic acidosis.
Charytan, 2019 [53]	Retrospective analysis in trials	4038 (591)	metformin vs. non-metformin among patients with diabetes and chronic kidney disease	NA	All-cause mortality: Overall: 0.49 (0.36–0.69) CKD S1–3: 0.61 (0.44–0.82) CKD S4–5: 0.83 (0.54–1.27) CV-death: Overall: 0.49 (0.32–0.74) CKD S1–3: 0.59 (0.38–0.9) CKD S4–5: 0.80 (0.46–1.39) ESKD: Overall: 1.01 (0.65–1.55) CKD S1–3: 0.70 (0.53–0.92) CKD S4–5: 0.95 (0.7–1.29)	Metformin might be safer for use in CKD than previously considered with reduced risk of death and cardiovascular events in individuals with stage 3 CKD.
Bergmark, 2019 [80]	Retrospective analysis in trials (2010–2013) FU: 2.1 years	12,156 (8971)	metformin vs. non-metformin among patients with diabetes and high CV risk	NA	All-cause mortality: 0.75 (0.59–0.95) CV-death: 0.68 (0.51–0.91) MI: 1.08 (0.83–1.41) Stroke: 1.07 (0.77–1.48) Hear failure: 1.23 (0.94–1.6)	Metformin use was associated with reduced risk of all-cause mortality, including after adjustment for clinical variables and biomarkers, but not lower rates of the composite end point of cardiovascular death, myocardial infarction, or ischemic stroke.
Roumie, 2019 [81]	Retrospective Cohort (2001–2016) FU: 1.1 year	174,882 metformin and sulfonylureas users	metformin vs. sulfonylureas	NA	MACE: Overall: 0.80 (0.75–0.86)	Among patients with diabetes and reduced kidney function persisting with monotherapy, treatment with metformin, compared with a sulfonylurea, was associated with a lower risk of MACE.
Hung, 2015 [82]	Retrospective Cohort (2000–2009) FU: 2.1 years	3252 (metformin 813)	metformin vs. non-metformin among patients with type 2 diabetes and stage 5 chronic kidney disease	Daily dose	All-cause mortality: 1.35 (1.2–1.51)	Use of metformin in people with type 2 diabetes and a serum creatinine concentration greater than 530 μmol/L was associated with an increased risk of all-cause mortality compared with non-users. Metformin use should not be encouraged in this patient group.
Ekstrom, 2012 [51]	Retrospective analysis in Swedish register (2004–2007) FU: 3.9 years	51,675 patients with type 2 diabetes	Metformin monotherapy vs. other GLDs	NA	All-cause mortality: Overall: 1.13 (1.01–1.27) Fatal/non-fatal CVD: Overall: 1.02 (0.93–1.12)	Metformin showed lower risk vs. insulin for CVD and all-cause mortality, and lower risk for all-cause mortality vs. other GLDs

Note: FU, follow-up; GLDs, glucose-lowering drugs; MACE, major adverse cardiovascular events; and ESKD, end-stage kidney disease.

The latest Kidney Disease Improving Global Outcomes (KDIGO) practice guideline recommended continuing use of metformin in CKD Stage 3b (eGFR: 30–44 mL/min/1.73 m^2^) with dose adjustments and increasing frequency of eGFR monitoring [83]. In both the USA and European Union (EU), according to the label, metformin can be used in CKD stage 3 (eGFR: 30–60 mL/min/1.73 m^2^), but not in stage 4 or stage 5. In the USA, metformin should not be initiated in CKD stage 3b, but may be continued in patients already treated with metformin [84]. Given the global burden of ESKD and the low cost of metformin, the safety and efficacy of metformin use in patients with CKD stage 3b and stage 4 should be further explored, preferably using RCT design due to a paucity of real-world data in these patients.

Metformin may protect the kidney via multiple mechanisms, such as reducing podocyte loss, tubulointerstitial injury, and mesangial cell dysfunction. Podocyte loss is the initiating event in the development of glomerular sclerosis in diabetic kidney disease (DKD). In animal models of T2D, metformin prevented podocyte loss via oxidative stress inhibition. In cultured podocytes, metformin reduced apoptosis via AMPK activation and inhibition of mTOR (mammalian target of rapamycin) activity [85]. In in vitro models, metformin attenuated palmitate-mediated mesangial apoptosis, ameliorated oxidative stress, and promoted autophagy. In high glucose-stimulated rat mesangial cells, metformin inhibited abnormal cell proliferation via the AMPK/SIRT1/forkhead box protein O1 (FOXO1) pathway [86]. Other studies demonstrated metformin in attenuating renal fibrosis in mice model of DKD by altering miR-192 expression [87]. Metformin also protected human epithelial cells against glucose-induced apoptosis by normalizing parkin protein expression and inducing mitophagy via repressing NF-KB expression [88].

More recently, beneficial effects of metformin have been shown in renal conditions other than DKD. Metformin was shown to inhibit cyst growth in patients with polycystic kidney disease (PKD) due to PKD1 mutation. In a zebrafish model, metformin inhibited cyst formation via activation of the AMPK pathway and modulated cellular events, such as autophagy, cellular proliferation, and inflammation [89].

### 3.2. Metformin and Infection

Metformin was originally introduced as an anti-influenza drug and had been proposed as an adjunct treatment in infective diseases [7]. During the current pandemic of COVID-19, there has been renewed interest in repurposing metformin as a host-directed adjunctive therapy to treat infections by altering the immune responses [37,90,91,92]. In this regard, metformin use was associated with a lower risk of death in patients with T2D affected by COVID-19 than their counterparts using other GLDs, especially among women with obesity [93,94]. Other studies indicated that metformin users who developed COVID-19 infection had lower levels of interleukin-6 (IL-6) [95] and other inflammatory markers than non-users [96]. Despite these observational data, definitive evidence from RCTs is lacking [92].

Patients with T2D are at high risk of pneumonia and other respiratory infections including chronic obstructive pulmonary diseases (COPD). In patients with community-acquired pneumonia, increased neutrophil-to-lymphocyte ratios indicating a heightened pro-inflammatory state had been associated with poor outcomes [13,97]. In a cohort of 3537 patients with T2D, long-term treatment with metformin was associated with reduced neutrophil-to-lymphocyte ratios, compared with sulfonylurea [98]. Previous studies reported a protective effect of metformin on pneumonia-related hospitalizations and pneumonia-related mortality among patients with T2D [99,100]. In an observational study of 36,990 patients aged >65 years with diabetes who were hospitalized with pneumonia, metformin users had a lower 30-day pneumonia-related mortality (odds ratio, OR = 0.80, 95% CI: 0.72–0.88) than non-users [100]. In a prospective diabetes register involving 15,784 patients with T2D in Hong Kong, metformin use was independently associated with lower incidence of pneumonia-related hospitalisation with a hazard ratio (HR) of 0.63 (95% CI: 0.52–0.77) and related-mortality (HR = 0.49, 95% CI: 0.33–0.73) adjusted for multiple confounders [101]. In a RCT comparing metformin vs. placebo for reducing adverse metabolic effects of glucocorticoids, in the analysis of the adverse events, metformin-treated patients had a lower incidence of pneumonia than the placebo group accompanied by lower levels of pro-inflammatory cytokines [102].

Several mechanisms have been proposed for the protective effects of metformin on pulmonary infections. In animal models of hyperoxia-induced lung injury, metformin reduced inflammatory cytokines such as IL-6 and TNF-α [103]. In other animal studies, metformin reduced the excessive release of neutrophil extracellular traps (NETs). The latter are extracellular DNA with anti-microbial actions although its overproduction may cause excessive inflammatory responses with deleterious consequences [104]. Metformin had been advocated as adjunctive therapy to improve outcomes in patients with sepsis [105]. In patients with tuberculosis, metformin had been shown to improve T-cell immunity and phagocytosis [106,107].

### 3.3. Metformin and Cancer

Diabetes, obesity, and cancer frequently coexist in part due to insulin resistance where excessive stimulation of the insulin/insulin-like growth factor (IGF-1) pathway might cause abnormal cell signaling and cancer growth [108]. Other epidemiological studies suggested additive risk associations of glycemic variability and burden with all-site cancer and cancer-related death in T2D [109,110]. In breast cancer cells, metformin exerted anticancer effects by changing the metabolic milieu and reducing the circulating insulin levels by improving insulin resistance with reduced insulin/IGF-I receptor-mediated phosphoinositide 3-kinases (PI3K) signaling [111].

**Figure 4:**
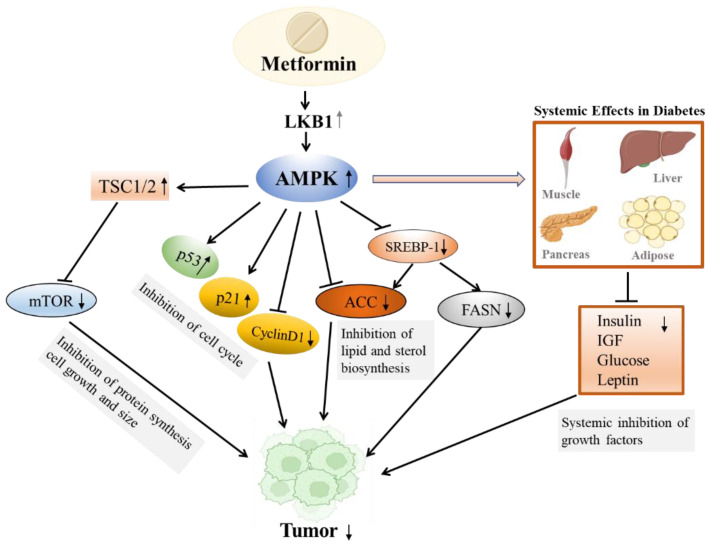
The action of metformin on cancer. Metformin activates adenosine monophosphate activated protein kinase (AMPK), an immediate downstream effector of the tumor suppressor liver kinase B1 (LKB1), resulting in inhibition of tumor growth. The various downstream effects of metformin-mediated AMPK activation in tumor growth inhibition include: (1) activation of the tuberous sclerosis complex (TSC) with inhibition of mammalian target of rapamycin (mTOR) activity, resulting in inhibition of protein synthesis and cell growth; (2) activation of p53 and p21 along with inhibition of cyclins, resulting in cell-cycle arrest; (3) inhibition of lipid and sterol biosynthetic pathways; (4) inhibition of sterol regulatory element-binding protein-1c (SREBP-1) by regulating its expression and phosphorylation, leading to down-regulation of fatty acid synthase (FASN) and acetyl-CoA carboxylase (ACC); (5) direct phosphorylation and inhibition of ACC; and (6) systemic effects on multiple organs such as reducing diabetes-associated cancers by improving glucose balance with reduced levels of growth factors such as insulin, insulin-like growth factor 1 (IGF-1) and leptin which can initiate and promote cancer growth with progression. Metformin had also been shown to reduce cancer events via AMPK-independent mechanisms [113,114] (adapted from reference [115,116]).

Metformin also inhibited mTOR pathway in cancer cells by activating AMPK and liver kinase B1 (LKB1) with reduced protein synthesis and cell growth [112]. In a systematic review, the Signal transducer and activator of transcription 3 (STAT3) was activated through the LKB1 and AMPK pathway which induced apoptosis in triple-negative breast cancer cells. Metformin had also been shown to influence the “sphingolipid rheostat”, shifting the balance away from Sphingosine-1-Phosphate towards ceramides with inhibition of cell growth and proliferation as demonstrated in an ovarian cancer cell line. Other anti-cancer mechanisms of metformin included increased fatty acids oxidation and reduced expression of transcription factors, such as specificity protein (Sp)1, Sp3, and Sp4 implicated in cancer growth [111]. Figure 4 summaries the main mechanisms for the action of metformin and cancer.

In experimental studies, metformin induced programmed cell death of cancer cells with inhibition of cell signals, such as vascular endothelial growth factor A (VEGFA), with reduced vascularization of tumor cells [36]. Metformin also modulated the immune response by activating anti-tumor T-cell activity [117]. By reducing oxygen and glucose consumption by tumor cells and increasing the intratumor oxygen levels, metformin sensitized patients’ responses to programmed death-ligand 1 (PD-L1) chemotherapy [118]. By altering the epigenetic signature of tumor cells, metformin also interfered with the signaling pathways that conferred chemoresistance of endometrial cancer cells and improved treatment responses to chemotherapy [119].

A large number of observational studies suggested an association of metformin use with reduced incidence of cancers [120]. In the Taiwan National Health Insurance Data Survey (2000–2007) including 12,005 metformin-users and 4597 non-metformin users, metformin use was associated with reduced risk of total, colorectal, liver and pancreatic cancer by up to 88% [121]. In a UK retrospective cohort of 62,809 patients, metformin monotherapy was associated with the lowest cancer risk, compared with insulin or sulfonylureas. Compared with metformin, sulfonylurea monotherapy was associated with a HR of 1.36 (95%CI: 1.19–1.54) for solid tumors (breast, colon, pancreas, and prostate cancer). The corresponding HR for combination therapy of metformin and sulfonylurea was 1.08 (95 %CI: 0.96–1.21) [122]. Other observational studies also reported low incidence of breast cancer among long-term metformin users vs. non-users [123]. In a database of health records from Tayside of Scotland, a comparative analysis between new metformin users and users of other medications showed consistently lower hazard for diagnosed cancer amongst metformin users [124].

In the Hong Kong Diabetes Register of 2658 patients with T2D free from cancer at enrolment [125], metformin use was associated with reduced risk of cancer in a dose-dependent manner. After adjusting for covariates, metformin non-users with high-density lipoprotein (HDL)-cholesterol <1.0 mmol/L had 5.8-fold increased hazards of cancer compared with metformin users with HDL-cholesterol ≥1.0 mmol/L [125]. In a post-hoc analysis of rosiglitazone-based RCTs, including the ADOPT (A Diabetes Outcome Progression Trial) and RECORD (Rosiglitazone Evaluated for Cardiovascular Outcomes and Regulation of Glycaemia in Diabetes) Trials [126], there was no difference in the incidence of cancers between patients treated with metformin vs. rosiglitazone. Apart from short duration of follow up, these studies were not powered to evaluate cancer incidence as a predefined endpoint [127]. Of note, these retrospective studies are subject to time-related biases, including immortal time bias and time-window bias, which might inflate the association between metformin and reduced risk of cancer [128]. Besides, it remained plausible that the risk differential might be due to increased cancer risk in the comparator group [129].

In a systematic review of 11 studies with accrual of 4042 cancer events and 529 cancer deaths, the researchers reported a 31% reduction in all-cancer risk amongst metformin-users compared with users of other GLDs (RR = 0.69, 95% CI: 0.61–0.79) [129]. The negative association was significant for pancreatic and hepatocellular cancer, and nonsignificant for colon, breast, and prostate cancer. On the other hand, in a pooled analysis of 9 RCTs including 821 patients with advanced or metastatic cancers from lung, breast, or pancreas as primary cancers, metformin did not improve tumor-related outcomes with a pooled OR of 1.23 (95% CI: 0.89–1.71) [130]. Similarly, metformin added to anticancer agents did not prolong progression-free survival (HR = 0.95, 95% CI: 0.75–1.21) or actual survival (HR = 0.97, 95% CI: 0.80–1.16) [130].

There are few RCTs that evaluated metformin with cancer as a predefined outcome measure. In a multi-center, double-blind, placebo-controlled phase 3 trial, nondiabetic adults who had resection of single or multiple colorectal adenomas or polyps were randomised to receive metformin 250 mg daily or placebo for 1 year. After 1 year, the total counts of polyps on colonoscopy was lower in the metformin group than the placebo group (relative risk (RR) = 0.67, 95% CI: 0.47–0.97). The corresponding RR for adenomas was 0.60 (95% CI: 0.39–0.92) [131].

In a phase 2 randomised trial, 40 women with metastatic breast cancer positive for estrogen receptor (ER)/progesterone receptor receiving chemotherapy were randomised to receive metformin (*n* = 22, mean age, 55 years) or placebo (*n* = 18, mean age, 57 years) for a mean of 151 days. The progression-free survival was 5.4 months in the metformin group vs. 6.3 months in the placebo group with a HR of 1.2 (95% CI: 0.63–2.31). The corresponding mean survival time were 20.2 and 24.2 months with a HR of 1.68 (95% CI: 0.79–3.55) suggesting that metformin did not confer survival benefits in patients with advanced disease although larger samples size and longer follow up period are needed to confirm these observations [132].

Obesity and hormonal dysregulation play important roles in the initiation and progression of some cancer events. Despite plausible mechanisms, the effects of metformin on hormone-responsive cancers, such as breast and endometrial cancers, remain to be clarified [133]. To this end, the NCIC Clinical Trials Group MA.32 had initiated a 5-year phase 3 RCT including 3649 women with early-stage breast cancer randomised to receive metformin 500 mg twice daily or placebo [134]. In an interim analysis at 6 months, metformin was associated with a tendency of increased insulin sensitivity, lower body mass index (BMI) [134], and reduced estradiol hormones vs. placebo [135].

### 3.4. Metformin and NAFLD

Metformin regulates cellular lipid and glucose metabolism by reducing mitochondrial oxidative processes resulting in activation of AMPK within the liver. This is accompanied by inhibition of de novo synthesis of fatty acids and increased β-oxidation of fatty acids leading to reduced liver steatosis. Metformin also reduced lipid accumulation by inhibiting differentiation of adipocytes with reduced production of adipokines [136]. In transgenic obese (ob/ob) mice with NAFLD, metformin reduced hepatic fat accumulation and liver steatosis and reversed hepatomegaly and abnormalities in liver enzymes [137]. In the high-fat-diet-induced NAFLD model of nondiabetic mice, metformin prevented and reversed liver steatosis and inflammation [138].

In the first open-label, pilot study, 20 non-diabetic patients with NAFLD were given metformin 500 mg thrice daily for 4 months but 6 patients did not adhere to treatment and were considered as control subjects. Insulin resistance was quantified using the euglycemic clamp technique and liver volume measurement by ultrasound scan. Metformin reduced the liver volume, moderately improved insulin sensitivity and normalized aminotransferase levels in 50% of the patients. Withdrawal of metformin was accompanied by a return of aminotransferase levels to the pre-treatment values. No changes in any of these parameters were observed in the control patients [139].

In a subsequent clinical trial using open-label, quasiexperimental design involving 28 overweight or obese patients with non-alcoholic steatohepatitis (NASH) treated with metformin 2000 mg daily for 12 months, the researchers reported improvement in insulin resistance, alanine transaminase (ALT), and histology [140]. In another comparative trial involving 34 patients with NASH treated with metformin 850 mg twice daily plus diet (*n* = 17) vs. diet alone (*n* = 17) for six months, the metformin group had reduction in ALT but no effects on histology [141].

Other researchers compared metformin vs. therapies, such as thiazolidinedione (TZDs) or vitamin E, in patients with NAFLD. In an open-label, randomised study, 55 non-diabetic patients with NAFLD were assigned to 12-month treatment with metformin 2000 mg daily (*n* = 55), vitamin E 800 IU daily (*n* = 28) or weight-reducing diet (*n* = 27). Liver enzymes and weight loss improved in all groups with the metformin group showing the highest odds of normalization of liver enzymes and metabolic profile. In a subgroup of 17 metformin users with 14 being non-responders, metformin use was associated with reduction in liver fat, necro-inflammation, and fibrosis although these results were limited by the small sample size [142]. In another open-label RCT comparing metformin, rosiglitazone, and combination therapy with both drugs, changes in liver enzymes and histology were observed only in patients treated with rosiglitazone or rosiglitazone plus metformin group but not in metformin alone group [143].

Following these initial encouraging results, subsequent double-blind placebo-controlled trials tended to report lack of benefits of metformin in patients with NAFLD. In one such trial, 48 patients with biopsy-proven NAFLD were randomised to receive either metformin or placebo for 6 months followed by repeat liver biopsy. Despite the positive effects on body weight, lipids, and glycaemic control, metformin treatment did not improve NAFLD score based on liver transaminases. No differences were observed in parameters of liver steatosis, assessed either histologically or by imaging [144]. Similarly, in two RCTs evaluating metformin (1000 to 1500 mg per day) vs. placebo in children with obesity and NAFLD, metformin did not improve liver histology, ALT and aspartate transaminase (AST) levels, BMI or insulin resistance [145]. In the Diabetes Prevention Program, participants with IGT randomised to metformin had lower ALT levels which was rendered non-significant once adjusted for weight loss [146]. Based on the latest systematic review of available data, there is insufficient evidence to support the use of metformin for relieving NAFLD or NASH [147].

### 3.5. Metformin and Cognitive Function

Ageing, T2D, and cognitive dysfunction frequently coexist. Apart from metabolic and vascular causes, Alzheimer’s disease (AD) characterized by deposition of amyloid-β (Aβ) plaques, neuroinflammation, neurofibrillary tangles, and neuronal loss is an important cause of dementia in patients with or without T2D. Experimental studies suggested that metformin might prevent amyloid plaque formation via AMPK-dependent pathways [148]. In part due to its anti-inflammatory effects, metformin improved microenvironment to promote neuro-glial cell survival and differentiation [149]. Additionally, metformin might directly influence the functional phenotype of microglia favoring a M2 phenotype which facilitated neural tissue repair following infarction [150]. In cultured astrocytes, metformin increased cellular consumption of oxygen and glucose with suppressed intermediates of amino acid and fatty acid metabolites but increased lactate production due to predominant anaerobic glycolysis. However, the clinical significance of these findings remained uncertain [151].

In animal studies, male Wistar rats treated with metformin at a dose of 100 mg/kg/day exhibited a reversal of scopolamine-induced cognitive impairment [152]. However, other studies using the same rat models reported no effect of metformin-fortified diet on cognitive function, despite improving insulin sensitivity [153]. In ischemic models, metformin improved neuron survival in dentate gyrus of diabetic mice [154]. In a rat model of forebrain ischaemia, metformin treatment for 7 days restored regulation of the AMPK/brain derived neurotrophic factor (BDNF)/70-kDa ribosomal protein S6 kinase (p70S6K) pathway with enhanced learning and memory [155].

Several observational studies investigated the effects of metformin on cognitive function [156] although many of these studies were limited by small sample size with incomplete documentation or adjustment for confounders, such as diabetes duration, glycaemic control, duration of metformin use, and comorbidities. One of the major confounders is the inhibitory effect of metformin on intestinal absorption of vitamin B12, especially with prolonged use in high dose where subclinical vitamin B12 deficiency had been associated with cognitive decline [157,158].

In a cross-sectional study of 4160 adults with normoglycemia (*n* = 1856), either with (*n* = 318) or without (*n* = 1986) metformin treatment, metformin use was associated with higher risk of vitamin B12 deficiency (odds ratio (OR) = 1.45, 95% CI: 1.03–2.02) and 1.3 times higher risk of cognitive impairment [159]. In a cross-sectional study conducted in Australia involving patients with AD and mild cognitive impairment [158], amongst patients with diabetes (*n* = 126), worse cognitive performance was associated with metformin use (OR = 2.23, 95% CI: 1.05–4.75), which was attenuated after adjustment for vitamin B12 levels (OR = 1.75, 95% CI: 0.81–3.78). On the other hand, in the Singapore Longitudinal Aging Study [160], the authors compared metformin use (*n* = 204) vs. non-use (*n* = 161) in diabetes patients with cognitive impairment (Mini-Mental State Exam ≤ 23). Metformin use was inversely associated with cognitive impairment prospectively (OR = 0.49, 95% CI: 0.25–0.95) with the lowest risk in those treated with metformin for 6 years or more [160].

Recently, in the prospective Sydney Memory and Ageing study including 1037 community-dwelling adults without dementia aged 70–90 years [161], participants underwent comprehensive neuropsychological testing every 2 years including executive function, visuospatial testing, and magnetic resonance imaging of brain to assess hippocampal volumes. Amongst 123 patients with T2D, metformin users had slower decline in global cognition and executive function. After adjustment for age, sex, BMI, smoking, blood pressure, and apolipoprotein E (APOE) genotypes, metformin use was associated with 81% lower risk of incident dementia than non-users [161].

There are few interventional studies of metformin on cognitive function and dementia. In a pilot, cross-over study including nondiabetic adults, a daily dose of 2000 mg metformin for 8 weeks was associated with favorable effects on executive function and measures related to learning, memory, and attention capacity [162]. In another placebo-controlled RCT involving 52 patients with T2D and depression, 6-month treatment with metformin was associated with improvements in cognitive function [163]. In the Diabetes Prevention Program, 2280 participants with IGT were randomised to receive metformin, placebo or lifestyle intervention for a mean of 2.3 years. In the 12–14 years post-randomisation period, metformin was associated with reduced risk of diabetes and lower plasma glucose with a neutral effect on cognitive outcomes [164]. Taken together, these results suggested that the effects of metformin on cognitive function depend on the dose and duration of metformin treatment. Although the overall evidence supports the favourable effects of metformin on cognitive function, this will need to be confirmed in larger and long-term interventional trials [165].

## 4. Conclusions

Type 2 diabetes is a global health challenge associated with multiple morbidities beyond cardiovascular–renal disease. The current COVID-19 pandemic highlighted the vulnerability of people with diabetes during acute infection due to their abnormal metabolic and proinflammatory milieu. With better control of cardiovascular risk factors, notably blood pressure and lipids, persistent hyperglycemia and obesity continue to give rise to other comorbidities including NAFLD, CKD, cancer, and cognitive dysfunction. Optimal energy metabolism is critical in maintaining cellular structure and function in any organism. Metformin inhibits mitochondrial respiratory chain, reduces ATP supply, and activates the AMP kinase to reduce excessive endogenous glucose production and energy storage in the form of protein and fat. These metabolic changes can improve insulin resistance, an important feature in type 2 diabetes. By restoring the energy balance, metformin can improve insulin action, glucose metabolism, and energy utilization at cellular levels. With better understanding on the pathogenetic roles of gut microbiota, there are emerging evidence supporting interactions between unabsorbed metformin and gut microbiota in reducing oxidative stress and inflammation which can trigger abnormal cell cycles in the host. These non-AMPK dependent mechanisms may contribute to the mitigating effects of metformin on cancer, infections, and age-related conditions, although further investigations are needed to confirm these hypotheses. Taken together, in patients with T2D, metformin forms a safe and efficacious low-cost base therapy for individualization of other GLDs. In patients without diabetes, the paucity of data especially RCTs limits the indications for use of metformin despite their potential benefits. Given the already extensive use of metformin in patients with T2D, large-scale RCTs in people without diabetes evaluating the effects of metformin on infection, cancer, and cognitive function will provide significant insights in our pursuit of reducing the burden of noncommunicable disease and aging-related co-morbidities.

## Data Availability

Data sharing not applicable.

## References

[1] Sun H., Saeedi P., Karuranga S., Pinkepank M., Ogurtsova K., Duncan B.B., Stein C., Basit A., Chan J.C.N., Mbanya J.C. (2021). IDF diabetes Atlas: Global, regional and country-level diabetes prevalence estimates for 2021 and projections for 2045. Diabetes Res. Clin. Pract..

[2] Gerich J.E. (2000). Physiology of glucose homeostasis. Diabetes Obes. Metab..

[3] Schrijvers B.F., De Vriese A.S., Flyvbjerg A. (2004). From hyperglycemia to diabetic kidney disease: The role of metabolic, hemodynamic, intracellular factors and growth factors/cytokines. Endocr. Rev..

[4] Tahrani A.A., Bailey C.J., Del Prato S., Barnett A.H. (2011). Management of type 2 diabetes: New and future developments in treatment. Lancet.

[5] American Diabetes Association (2021). 9. Pharmacologic Approaches to Glycemic Treatment: Standards of Medical Care in Diabetes-2021. Diabetes Care.

[6] Marshall S.M. (2017). 60 years of metformin use: A glance at the past and a look to the future. Diabetologia.

[7] Bailey C.J. (2017). Metformin: Historical overview. Diabetologia.

[8] Cetin M., Sahin S. (2016). Microparticulate and nanoparticulate drug delivery systems for metformin hydrochloride. Drug Deliv..

[9] Gormsen L.C., Sundelin E.I., Jensen J.B., Vendelbo M.H., Jakobsen S., Munk O.L., Hougaard Christensen M.M., Brøsen K., Frøkiær J., Jessen N. (2016). In Vivo Imaging of Human 11C-Metformin in Peripheral Organs: Dosimetry, Biodistribution, and Kinetic Analyses. J. Nucl. Med..

[10] Bailey C.J., Wilcock C., Scarpello J.H. (2008). Metformin and the intestine. Diabetologia.

[11] Chen Y., Shan X., Luo C., He Z. (2020). Emerging nanoparticulate drug delivery systems of metformin. J. Pharm. Investig..

[12] LaMoia T.E., Shulman G.I. (2020). Cellular and Molecular Mechanisms of Metformin Action. Endocr. Rev..

[13] Rena G., Hardie D.G., Pearson E.R. (2017). The mechanisms of action of metformin. Diabetologia.

[14] Madiraju A.K., Erion D.M., Rahimi Y., Zhang X.-M., Braddock D.T., Albright R.A., Prigaro B.J., Wood J.L., Bhanot S., Macdonald M.J. (2014). Metformin suppresses gluconeogenesis by inhibiting mitochondrial glycerophosphate dehydrogenase. Nature.

[15] Pernicova I., Korbonits M. (2014). Metformin—mode of action and clinical implications for diabetes and cancer. Nat. Rev. Endocrinol..

[16] Li M., Li X., Zhang H., Lu Y. (2018). Molecular Mechanisms of Metformin for Diabetes and Cancer Treatment. Front. Physiol..

[17] Song A., Zhang C., Meng X. (2021). Mechanism and application of metformin in kidney diseases: An update. Biomed. Pharmacother..

[18] Jaune E., Rocchi S. (2018). Metformin: Focus on Melanoma. Front. Endocrinol..

[19] Ma R., Yi B., Riker A.I., Xi Y. (2020). Metformin and cancer immunity. Acta Pharmacol. Sin..

[20] Giaccari A., Solini A., Frontoni S., Del Prato S. (2021). Metformin Benefits: Another Example for Alternative Energy Substrate Mechanism?. Diabetes Care.

[21] Salpeter S.R., Greyber E., Pasternak G.A., Salpeter E.E. (2003). Risk of Fatal and Nonfatal Lactic Acidosis With Metformin Use in Type 2 Diabetes Mellitus. Arch. Intern. Med..

[22] Lipska K.J., Yao X., Herrin J., McCoy R.G., Ross J.S., Steinman M.A., Inzucchi S.E., Gill T.M., Krumholz H.M., Shah N.D. (2017). Trends in Drug Utilization, Glycemic Control, and Rates of Severe Hypoglycemia, 2006–2013. Diabetes Care.

[23] Yang A., Wu H., Lau E.S., Ma R.C., Kong A.P., So W.Y., Luk A.O., Chan J.C., Chow E. (2020). Trends in Glucose-Lowering Drug Use, Glycemic Control, and Severe Hypoglycemia in Adults With Diabetes in Hong Kong, 2002–2016. Diabetes Care.

[24] Harris S.T., Patorno E., Zhuo M., Kim S.C., Paik J.M. (2021). Prescribing Trends of Antidiabetes Medications in Patients With Type 2 Diabetes and Diabetic Kidney Disease, a Cohort Study. Diabetes Care.

[25] Ala M., Ala M. (2021). Metformin for Cardiovascular Protection, Inflammatory Bowel Disease, Osteoporosis, Periodontitis, Polycystic Ovarian Syndrome, Neurodegeneration, Cancer, Inflammation and Senescence: What Is Next?. ACS Pharmacol. Transl. Sci..

[26] Foretz M., Hébrard S., Leclerc J., Zarrinpashneh E., Soty M., Mithieux G., Sakamoto K., Andreelli F., Viollet B. (2010). Metformin inhibits hepatic gluconeogenesis in mice independently of the LKB1/AMPK pathway via a decrease in hepatic energy state. J. Clin. Investig..

[27] Lv Z., Guo Y. (2020). Metformin and its benefits for various diseases. Front. Endocrinol..

[28] Gurung M., Li Z., You H., Rodrigues R., Jump D.B., Morgun A., Shulzhenko N. (2020). Role of gut microbiota in type 2 diabetes pathophysiology. EBioMedicine.

[29] Wu H., Esteve E., Tremaroli V., Khan M.T., Caesar R., Mannerås-Holm L., Ståhlman M., Olsson L.M., Serino M., Planas-Fèlix M. (2017). Metformin alters the gut microbiome of individuals with treatment-naive type 2 diabetes, contributing to the therapeutic effects of the drug. Nat. Med..

[30] Vallianou N.G., Stratigou T., Tsagarakis S. (2019). Metformin and gut microbiota: Their interactions and their impact on diabetes. Hormones.

[31] De La Cuesta-Zuluaga J., Mueller N.T., Corrales-Agudelo V., Velásquez-Mejía E.P., Carmona J.A., Abad J.M., Escobar J.S. (2017). Metformin is associated with higher relative abundance of mucin-degrading Akkermansia muciniphila and several short-chain fatty acid–producing microbiota in the gut. Diabetes Care.

[32] Turroni F., Milani C., Duranti S., Mancabelli L., Mangifesta M., Viappiani A., Lugli G.A., Ferrario C., Gioiosa L., Ferrarini A. (2016). Deciphering bifidobacterial-mediated metabolic interactions and their impact on gut microbiota by a multi-omics approach. ISME J..

[33] Jeon B.S., Choi O., Um Y., Sang B.-I. (2016). Production of medium-chain carboxylic acids by Megasphaera sp. MH with supplemental electron acceptors. Biotechnol. Biofuels.

[34] Sun L., Xie C., Wang G., Wu Y., Wu Q., Wang X., Liu J., Deng Y., Xia J., Chen B. (2018). Gut microbiota and intestinal FXR mediate the clinical benefits of metformin. Nat. Med..

[35] Cabreiro F., Au C., Leung K.Y., Vergara-Irigaray N., Cochemé H.M., Noori T., Weinkove D., Schuster E., Greene N.D., Gems D. (2013). Metformin retards aging in C. elegans by altering microbial folate and methionine metabolism. Cell.

[36] Hsu S.-K., Cheng K.-C., Mgbeahuruike M.O., Lin Y.-H., Wu C.-Y., Wang H.-M.D., Yen C.-H., Chiu C.-C., Sheu S.-J. (2021). New Insight into the Effects of Metformin on Diabetic Retinopathy, Aging and Cancer: Nonapoptotic Cell Death, Immunosuppression, and Effects beyond the AMPK Pathway. Int. J. Mol. Sci..

[37] Kristófi R., Eriksson J.W. (2021). Metformin as an anti-inflammatory agent: A short review. J. Endocrinol..

[38] Holman R.R., Paul S.K., Bethel M.A., Matthews D.R., Neil H.A. (2008). 10-year follow-up of intensive glucose control in type 2 diabetes. N. Engl. J. Med..

[39] Aroda V.R., Knowler W.C., Crandall J.P., Perreault L., Edelstein S.L., Jeffries S.L., Molitch M.E., Pi-Sunyer X., Darwin C., Heckman-Stoddard B.M. (2017). Metformin for diabetes prevention: Insights gained from the Diabetes Prevention Program/Diabetes Prevention Program Outcomes Study. Diabetologia.

[40] Griffin S.J., Leaver J.K., Irving G.J. (2017). Impact of metformin on cardiovascular disease: A meta-analysis of randomised trials among people with type 2 diabetes. Diabetologia.

[41] Zhang K., Yang W., Dai H., Deng Z. (2020). Cardiovascular risk following metformin treatment in patients with type 2 diabetes mellitus: Results from meta-analysis. Diabetes Res. Clin. Pract..

[42] Han Y., Xie H., Liu Y., Gao P., Yang X., Shen Z. (2019). Effect of metformin on all-cause and cardiovascular mortality in patients with coronary artery diseases: A systematic review and an updated meta-analysis. Cardiovasc. Diabetol..

[43] Campbell J.M., Bellman S.M., Stephenson M.D., Lisy K. (2017). Metformin reduces all-cause mortality and diseases of ageing independent of its effect on diabetes control: A systematic review and meta-analysis. Ageing Res. Rev..

[44] Li X., Celotto S., Pizzol D., Gasevic D., Ji M.M., Barnini T., Solmi M., Stubbs B., Smith L., López Sánchez G.F. (2021). Metformin and health outcomes: An umbrella review of systematic reviews with meta-analyses. Eur. J. Clin. Investig..

[45] Monami M., Candido R., Pintaudi B., Targher G., Mannucci E. (2021). Effect of metformin on all-cause mortality and major adverse cardiovascular events: An updated meta-analysis of randomized controlled trials. Nutr. Metab Cardiovasc. Dis..

[46] Raee M.R., Nargesi A.A., Heidari B., Mansournia M.A., Larry M., Rabizadeh S., Zarifkar M., Esteghamati A., Nakhjavani M. (2017). All-Cause and Cardiovascular Mortality following Treatment with Metformin or Glyburide in Patients with Type 2 Diabetes Mellitus. Arch. Iran. Med..

[47] Scheller N.M., Mogensen U.M., Andersson C., Vaag A., Torp-Pedersen C. (2014). All-cause mortality and cardiovascular effects associated with the DPP-IV inhibitor sitagliptin compared with metformin, a retrospective cohort study on the Danish population. Diabetes Obes. Metab..

[48] Roumie C.L., Hung A.M., Greevy R.A., Grijalva C.G., Liu X., Murff H.J., Elasy T.A., Griffin M.R. (2012). Comparative effectiveness of sulfonylurea and metformin monotherapy on cardiovascular events in type 2 diabetes mellitus: A cohort study. Ann. Intern. Med..

[49] Roumie C.L., Min J.Y., D’Agostino McGowan L., Presley C., Grijalva C.G., Hackstadt A.J., Hung A.M., Greevy R.A., Elasy T., Griffin M.R. (2017). Comparative Safety of Sulfonylurea and Metformin Monotherapy on the Risk of Heart Failure: A Cohort Study. J. Am. Heart Assoc..

[50] Johnson J.A., Majumdar S.R., Simpson S.H., Toth E.L. (2002). Decreased mortality associated with the use of metformin compared with sulfonylurea monotherapy in type 2 diabetes. Diabetes Care.

[51] Ekström N., Schiöler L., Svensson A.M., Eeg-Olofsson K., Jonasson J.M., Zethelius B., Cederholm J., Eliasson B., Gudbjörnsdottir S. (2012). Effectiveness and safety of metformin in 51,675 patients with type 2 diabetes and different levels of renal function: A cohort study from the Swedish National Diabetes Register. BMJ Open.

[52] Pantalone K.M., Kattan M.W., Yu C., Wells B.J., Arrigain S., Jain A., Atreja A., Zimmerman R.S. (2012). Increase in overall mortality risk in patients with type 2 diabetes receiving glipizide, glyburide or glimepiride monotherapy versus metformin: A retrospective analysis. Diabetes Obes. Metab..

[53] Charytan D.M., Solomon S.D., Ivanovich P., Remuzzi G., Cooper M.E., McGill J.B., Parving H.H., Parfrey P., Singh A.K., Burdmann E.A. (2019). Metformin use and cardiovascular events in patients with type 2 diabetes and chronic kidney disease. Diabetes Obes. Metab..

[54] Cheng Y.Y., Leu H.B., Chen T.J., Chen C.L., Kuo C.H., Lee S.D., Kao C.L. (2014). Metformin-inclusive therapy reduces the risk of stroke in patients with diabetes: A 4-year follow-up study. J. Stroke Cerebrovasc. Dis..

[55] Mogensen U.M., Andersson C., Fosbøl E.L., Schramm T.K., Vaag A., Scheller N.M., Torp-Pedersen C., Gislason G., Køber L. (2015). Sulfonylurea in combination with insulin is associated with increased mortality compared with a combination of insulin and metformin in a retrospective Danish nationwide study. Diabetologia.

[56] Evans J.M., Ogston S.A., Emslie-Smith A., Morris A.D. (2006). Risk of mortality and adverse cardiovascular outcomes in type 2 diabetes: A comparison of patients treated with sulfonylureas and metformin. Diabetologia.

[57] Sillars B., Davis W.A., Hirsch I.B., Davis T.M. (2010). Sulphonylurea-metformin combination therapy, cardiovascular disease and all-cause mortality: The Fremantle Diabetes Study. Diabetes Obes. Metab..

[58] Morgan C.L., Mukherjee J., Jenkins-Jones S., Holden S.E., Currie C.J. (2014). Association between first-line monotherapy with sulphonylurea versus metformin and risk of all-cause mortality and cardiovascular events: A retrospective, observational study. Diabetes Obes. Metab..

[59] Breunig I.M., Shaya F.T., McPherson M.L., Snitker S. (2014). Development of heart failure in Medicaid patients with type 2 diabetes treated with pioglitazone, rosiglitazone, or metformin. J. Manag. Care Spec. Pharm..

[60] Fung C.S., Wan E.Y., Wong C.K., Jiao F., Chan A.K. (2015). Effect of metformin monotherapy on cardiovascular diseases and mortality: A retrospective cohort study on Chinese type 2 diabetes mellitus patients. Cardiovasc. Diabetol..

[61] Mather K.J., Verma S., Anderson T.J. (2001). Improved endothelial function with metformin in type 2 diabetes mellitus. J. Am. Coll. Cardiol..

[62] de Jager J., Kooy A., Schalkwijk C., van der Kolk J., Lehert P., Bets D., Wulffelé M.G., Donker A.J., Stehouwer C.D. (2014). Long-term effects of metformin on endothelial function in type 2 diabetes: A randomized controlled trial. J. Intern. Med..

[63] Triggle C.R., Ding H. (2017). Metformin is not just an antihyperglycaemic drug but also has protective effects on the vascular endothelium. Acta Physiol..

[64] Davis B.J., Xie Z., Viollet B., Zou M.H. (2006). Activation of the AMP-activated kinase by antidiabetes drug metformin stimulates nitric oxide synthesis in vivo by promoting the association of heat shock protein 90 and endothelial nitric oxide synthase. Diabetes.

[65] Yu J.W., Deng Y.P., Han X., Ren G.F., Cai J., Jiang G.J. (2016). Metformin improves the angiogenic functions of endothelial progenitor cells via activating AMPK/eNOS pathway in diabetic mice. Cardiovasc. Diabetol..

[66] An H., Wei R., Ke J., Yang J., Liu Y., Wang X., Wang G., Hong T. (2016). Metformin attenuates fluctuating glucose-induced endothelial dysfunction through enhancing GTPCH1-mediated eNOS recoupling and inhibiting NADPH oxidase. J. Diabetes Complicat..

[67] Nafisa A., Gray S.G., Cao Y., Wang T., Xu S., Wattoo F.H., Barras M., Cohen N., Kamato D., Little P.J. (2018). Endothelial function and dysfunction: Impact of metformin. Pharmacol. Ther..

[68] Zheng Z., Chen H., Li J., Li T., Zheng B., Zheng Y., Jin H., He Y., Gu Q., Xu X. (2012). Sirtuin 1-mediated cellular metabolic memory of high glucose via the LKB1/AMPK/ROS pathway and therapeutic effects of metformin. Diabetes.

[69] Marfella R., Acampora R., Verrazzo G., Ziccardi P., De Rosa N., Giunta R., Giugliano D. (1996). Metformin improves hemodynamic and rheological responses to L-arginine in NIDDM patients. Diabetes Care.

[70] Grant P.J. (2003). Beneficial effects of metformin on haemostasis and vascular function in man. Diabetes Metab..

[71] Grant P.J., Stickland M.H., Booth N.A., Prentice C.R. (1991). Metformin causes a reduction in basal and post-venous occlusion plasminogen activator inhibitor-1 in type 2 diabetic patients. Diabet. Med..

[72] Xin G., Wei Z., Ji C., Zheng H., Gu J., Ma L., Huang W., Morris-Natschke S.L., Yeh J.L., Zhang R. (2016). Metformin Uniquely Prevents Thrombosis by Inhibiting Platelet Activation and mtDNA Release. Sci. Rep..

[73] Cabrera C.S., Lee A.S., Olsson M., Schnecke V., Westman K., Lind M., Greasley P.J., Skrtic S. (2020). Impact of CKD Progression on Cardiovascular Disease Risk in a Contemporary UK Cohort of Individuals With Diabetes. Kidney Int. Rep..

[74] Petrie J.R., Rossing P.R., Campbell I.W. (2020). Metformin and cardiorenal outcomes in diabetes: A reappraisal. Diabetes Obes. Metab..

[75] Gansevoort R.T., Correa-Rotter R., Hemmelgarn B.R., Jafar T.H., Heerspink H.J., Mann J.F., Matsushita K., Wen C.P. (2013). Chronic kidney disease and cardiovascular risk: Epidemiology, mechanisms, and prevention. Lancet.

[76] Phung O.J., Scholle J.M., Talwar M., Coleman C.I. (2010). Effect of noninsulin antidiabetic drugs added to metformin therapy on glycemic control, weight gain, and hypoglycemia in type 2 diabetes. JAMA.

[77] Neven E., Vervaet B., Brand K., Gottwald-Hostalek U., Opdebeeck B., De Maré A., Verhulst A., Lalau J.D., Kamel S., De Broe M.E. (2018). Metformin prevents the development of severe chronic kidney disease and its associated mineral and bone disorder. Kidney Int..

[78] Whitlock R.H., Hougen I., Komenda P., Rigatto C., Clemens K.K., Tangri N. (2020). A Safety Comparison of Metformin vs Sulfonylurea Initiation in Patients With Type 2 Diabetes and Chronic Kidney Disease: A Retrospective Cohort Study. Mayo Clin. Proc..

[79] Kwon S., Kim Y.C., Park J.Y., Lee J., An J.N., Kim C.T., Oh S., Park S., Kim D.K., Oh Y.K. (2020). The Long-term Effects of Metformin on Patients with Type 2 Diabetic Kidney Disease. Diabetes Care.

[80] Bergmark B.A., Bhatt D.L., McGuire D.K., Cahn A., Mosenzon O., Steg P.G., Im K., Kanevsky E., Gurmu Y., Raz I. (2019). Metformin Use and Clinical Outcomes among Patients with Diabetes Mellitus with or Without Heart Failure or Kidney Dysfunction: Observations from the SAVOR-TIMI 53 Trial. Circulation.

[81] Roumie C.L., Chipman J., Min J.Y., Hackstadt A.J., Hung A.M., Greevy R.A., Grijalva C.G., Elasy T., Griffin M.R. (2019). Association of Treatment With Metformin vs Sulfonylurea With Major Adverse Cardiovascular Events Among Patients With Diabetes and Reduced Kidney Function. JAMA.

[82] Hung S.C., Chang Y.K., Liu J.S., Kuo K.L., Chen Y.H., Hsu C.C., Tarng D.C. (2015). Metformin use and mortality in patients with advanced chronic kidney disease: National, retrospective, observational, cohort study. Lancet Diabetes Endocrinol..

[83] Navaneethan S.D., Zoungas S., Caramori M.L., Chan J.C.N., Heerspink H.J.L., Hurst C., Liew A., Michos E.D., Olowu W.A., Sadusky T. (2020). Diabetes Management in Chronic Kidney Disease: Synopsis of the 2020 KDIGO Clinical Practice Guideline. Ann. Intern. Med..

[84] Buse J.B., Wexler D.J., Tsapas A., Rossing P., Mingrone G., Mathieu C., D’Alessio D.A., Davies M.J. (2020). 2019 Update to: Management of Hyperglycemia in Type 2 Diabetes, 2018. A Consensus Report by the American Diabetes Association (ADA) and the European Association for the Study of Diabetes (EASD). Diabetes Care.

[85] Kim J., Shon E., Kim C.S., Kim J.S. (2012). Renal podocyte injury in a rat model of type 2 diabetes is prevented by metformin. Exp. Diabetes Res..

[86] Kim D.I., Park M.J., Heo Y.R., Park S.H. (2015). Metformin ameliorates lipotoxicity-induced mesangial cell apoptosis partly via upregulation of glucagon like peptide-1 receptor (GLP-1R). Arch. Biochem. Biophys..

[87] Yu S., Zhao H., Yang W., Amat R., Peng J., Li Y., Deng K., Mao X., Jiao Y. (2019). The alcohol extract of Coreopsis tinctoria Nutt ameliorates diabetes and diabetic nephropathy in db/db mice through miR-192/miR-200b and PTEN/AKT and ZEB2/ECM pathways. BioMed Res. Int..

[88] Zhao Y., Sun M. (2020). Metformin rescues Parkin protein expression and mitophagy in high glucose-challenged human renal epithelial cells by inhibiting NF-κB via PP2A activation. Life Sci..

[89] Chang M.Y., Ma T.L., Hung C.C., Tian Y.C., Chen Y.C., Yang C.W., Cheng Y.C. (2017). Metformin Inhibits Cyst Formation in a Zebrafish Model of Polycystin-2 Deficiency. Sci. Rep..

[90] Sharma S., Ray A., Sadasivam B. (2020). Metformin in COVID-19: A possible role beyond diabetes. Diabetes Res. Clin. Pract..

[91] Dalan R. (2020). Metformin, neutrophils and COVID-19 infection. Diabetes Res. Clin. Pract..

[92] Samuel S.M., Varghese E., Büsselberg D. (2021). Therapeutic Potential of Metformin in COVID-19: Reasoning for Its Protective Role. Trends Microbiol..

[93] Bramante C.T., Ingraham N.E., Murray T.A., Marmor S., Hovertsen S., Gronski J., McNeil C., Feng R., Guzman G., Abdelwahab N. (2021). Metformin and risk of mortality in patients hospitalised with COVID-19: A retrospective cohort analysis. Lancet Healthy Longev..

[94] Poly T.N., Islam M.M., Li Y.J., Lin M.C., Hsu M.H., Wang Y.C. (2021). Metformin Use Is Associated with Decreased Mortality in COVID-19 Patients with Diabetes: Evidence from Retrospective Studies and Biological Mechanism. J. Clin. Med..

[95] Chen Y., Yang D., Cheng B., Chen J., Peng A., Yang C., Liu C., Xiong M., Deng A., Zhang Y. (2020). Clinical Characteristics and Outcomes of Patients With Diabetes and COVID-19 in Association With Glucose-Lowering Medication. Diabetes Care.

[96] Cheng X., Liu Y.-M., Li H., Zhang X., Lei F., Qin J.-J., Chen Z., Deng K.-Q., Lin L., Chen M.-M. (2020). Metformin Use Is Associated with Increased Incidence of Acidosis but not Mortality in Individuals with COVID-19 and Pre-existing Type 2 Diabetes. Cell Metab..

[97] De Jager C.P., Wever P.C., Gemen E.F., Kusters R., van Gageldonk-Lafeber A.B., van der Poll T., Laheij R.J. (2012). The neutrophil-lymphocyte count ratio in patients with community-acquired pneumonia. PLoS ONE.

[98] Cameron A.R., Morrison V.L., Levin D., Mohan M., Forteath C., Beall C., McNeilly A.D., Balfour D.J., Savinko T., Wong A.K. (2016). Anti-inflammatory effects of metformin irrespective of diabetes status. Circ. Res..

[99] Mor A., Petersen I., Sørensen H.T., Thomsen R.W. (2016). Metformin and other glucose-lowering drug initiation and rates of community-based antibiotic use and hospital-treated infections in patients with type 2 diabetes: A Danish nationwide population-based cohort study. BMJ Open.

[100] Mortensen E., Anzueto A. (2018). Association of metformin and mortality for patients with diabetes who are hospitalized with pneumonia. Eur. Respir. J..

[101] Yang A., Shi M., Wu H., Lau E.S.H., Ma R.C.W., Kong A.P.S., So W.Y., Luk A.O.Y., Chan J.C.N., Chow E. (2021). Long-term metformin use and risk of pneumonia and related death in type 2 diabetes: A registry-based cohort study. Diabetologia.

[102] Pernicova I., Kelly S., Ajodha S., Sahdev A., Bestwick J.P., Gabrovska P., Akanle O., Ajjan R., Kola B., Stadler M. (2020). Metformin to reduce metabolic complications and inflammation in patients on systemic glucocorticoid therapy: A randomised, double-blind, placebo-controlled, proof-of-concept, phase 2 trial. Lancet Diabetes Endocrinol..

[103] Chen X., Walther F.J., Sengers R.M., Laghmani E.H., Salam A., Folkerts G., Pera T., Wagenaar G.T. (2015). Metformin attenuates hyperoxia-induced lung injury in neonatal rats by reducing the inflammatory response. Am. J. Physiol. Lung Cell Mol. Physiol..

[104] Menegazzo L., Scattolini V., Cappellari R., Bonora B.M., Albiero M., Bortolozzi M., Romanato F., Ceolotto G., de Kreutzeberg S.V., Avogaro A. (2018). The antidiabetic drug metformin blunts NETosis in vitro and reduces circulating NETosis biomarkers in vivo. Acta Diabetol..

[105] Yu X., Li L., Xia L., Feng X., Chen F., Cao S., Wei X. (2019). Impact of metformin on the risk and treatment outcomes of tuberculosis in diabetics: A systematic review. BMC Infect. Dis..

[106] Singhal A., Jie L., Kumar P., Hong G.S., Leow M.K.-S., Paleja B., Tsenova L., Kurepina N., Chen J., Zolezzi F. (2014). Metformin as adjunct antituberculosis therapy. Sci. Transl. Med..

[107] Rodriguez-Carlos A., Valdez-Miramontes C., Marin-Luevano P., González-Curiel I., Enciso-Moreno J.A., Rivas-Santiago B. (2020). Metformin promotes Mycobacterium tuberculosis killing and increases the production of human β-defensins in lung epithelial cells and macrophages. Microbes. Infect..

[108] Yang X., Lee H.M., Chan J.C. (2015). Drug-subphenotype interactions for cancer in type 2 diabetes mellitus. Nat. Rev. Endocrinol..

[109] Mao D., Lau E.S.H., Wu H., Yang A., Shi M., Fan B., Tam C., Chow E., Kong A.P.S., Ma R.C.W. (2022). Risk associations of long-term HbA1c variability and obesity on cancer events and cancer-specific death in 15,286 patients with diabetes—A prospective cohort study. Lancet Reg. Health—West. Pac..

[110] Mao D., Lau E.S.H., Wu H., Yang A., Shi M., Fan B., Tam C., Chow E., Kong A.P.S., Ma R.C.W. (2022). Risk associations of glycemic burden and obesity with liver cancer—A 10-year analysis of 16,410 patients with type 2 diabetes. Hepatol. Commun..

[111] Vancura A., Bu P., Bhagwat M., Zeng J., Vancurova I. (2018). Metformin as an Anticancer Agent. Trends Pharmacol. Sci..

[112] Heckman-Stoddard B.M., Decensi A., Sahasrabuddhe V.V., Ford L.G. (2017). Repurposing metformin for the prevention of cancer and cancer recurrence. Diabetologia.

[113] Ben Sahra I., Regazzetti C., Robert G., Laurent K., Le Marchand-Brustel Y., Auberger P., Tanti J.F., Giorgetti-Peraldi S., Bost F. (2011). Metformin, independent of AMPK, induces mTOR inhibition and cell-cycle arrest through REDD1. Cancer Res..

[114] Rattan R., Giri S., Hartmann L.C., Shridhar V. (2011). Metformin attenuates ovarian cancer cell growth in an AMP-kinase dispensable manner. J. Cell Mol. Med..

[115] Rattan R., Ali Fehmi R., Munkarah A. (2012). Metformin: An emerging new therapeutic option for targeting cancer stem cells and metastasis. J. Oncol..

[116] He H., Ke R., Lin H., Ying Y., Liu D., Luo Z. (2015). Metformin, an old drug, brings a new era to cancer therapy. Cancer J..

[117] Xiong Y., Song W., Shen L., Wang Y., Zhang J., Hu M., Liu Y., Li J., Musetti S., Liu R. (2020). Oral Metformin and Polymetformin Reprogram Immunosuppressive Microenvironment and Boost Immune Checkpoint Inhibitor Therapy in Colorectal Cancer. Adv. Ther..

[118] Scharping N.E., Menk A.V., Whetstone R.D., Zeng X., Delgoffe G.M. (2017). Efficacy of PD-1 Blockade Is Potentiated by Metformin-Induced Reduction of Tumor Hypoxia. Cancer Immunol. Res..

[119] Bai M., Yang L., Liao H., Liang X., Xie B., Xiong J., Tao X., Chen X., Cheng Y., Chen X. (2018). Metformin sensitizes endometrial cancer cells to chemotherapy through IDH1-induced Nrf2 expression via an epigenetic mechanism. Oncogene.

[120] Mallik R., Chowdhury T.A. (2018). Metformin in cancer. Diabetes Res. Clin. Pract..

[121] Lee M.-S., Hsu C.-C., Wahlqvist M.L., Tsai H.-N., Chang Y.-H., Huang Y.-C. (2011). Type 2 diabetes increases and metformin reduces total, colorectal, liver and pancreatic cancer incidences in Taiwanese: A representative population prospective cohort study of 800,000 individuals. BMC Cancer.

[122] Currie C.J., Poole C.D., Gale E.A.M. (2009). The influence of glucose-lowering therapies on cancer risk in type 2 diabetes. Diabetologia.

[123] Bodmer M., Meier C., Krahenbuhl S., Jick S.S., Meier C.R. (2010). Long-Term Metformin Use Is Associated With Decreased Risk of Breast Cancer. Diabetes Care.

[124] Libby G., Donnelly L.A., Donnan P.T., Alessi D.R., Morris A.D., Evans J.M.M. (2009). New Users of Metformin Are at Low Risk of Incident Cancer: A cohort study among people with type 2 diabetes. Diabetes Care.

[125] Yang X., So W.Y., Ma R.C.W., Kong A.P.S., Lee H.M., Yu L.W.L., Chow C.C., Ozaki R., Ko G.T.C., Chan J.C.N. (2011). Low HDL Cholesterol, Metformin Use, and Cancer Risk in Type 2 Diabetes: The Hong Kong Diabetes Registry. Diabetes Care.

[126] Viberti G., Kahn S.E., Greene D.A., Herman W.H., Zinman B., Holman R.R., Haffner S.M., Levy D., Lachin J.M., Berry R.A. (2002). A diabetes outcome progression trial (ADOPT): An international multicenter study of the comparative efficacy of rosiglitazone, glyburide, and metformin in recently diagnosed type 2 diabetes. Diabetes Care.

[127] Home P.D., Kahn S.E., Jones N.P., Noronha D., Beck-Nielsen H., Viberti G. (2010). Experience of malignancies with oral glucose-lowering drugs in the randomised controlled ADOPT (A Diabetes Outcome Progression Trial) and RECORD (Rosiglitazone Evaluated for Cardiovascular Outcomes and Regulation of Glycaemia in Diabetes) clinical trials. Diabetologia.

[128] Suissa S., Azoulay L. (2012). Metformin and the risk of cancer: Time-related biases in observational studies. Diabetes Care.

[129] Decensi A., Puntoni M., Goodwin P., Cazzaniga M., Gennari A., Bonanni B., Gandini S. (2010). Metformin and Cancer Risk in Diabetic Patients: A Systematic Review and Meta-analysis. Cancer Prev. Res..

[130] Kim H.S., Kim J.H., Jang H.J., Lee J. (2020). The addition of metformin to systemic anticancer therapy in advanced or metastatic cancers: A meta-analysis of randomized controlled trials. Int. J. Med. Sci..

[131] Higurashi T., Hosono K., Takahashi H., Komiya Y., Umezawa S., Sakai E., Uchiyama T., Taniguchi L., Hata Y., Uchiyama S. (2016). Metformin for chemoprevention of metachronous colorectal adenoma or polyps in post-polypectomy patients without diabetes: A multicentre double-blind, placebo-controlled, randomised phase 3 trial. Lancet Oncol..

[132] Pimentel I., Lohmann A.E., Ennis M., Dowling R.J.O., Cescon D., Elser C., Potvin K.R., Haq R., Hamm C., Chang M.C. (2019). A phase II randomized clinical trial of the effect of metformin versus placebo on progression-free survival in women with metastatic breast cancer receiving standard chemotherapy. Breast.

[133] Farkhondeh T., Amirabadizadeh A., Aramjoo H., Llorens S., Roshanravan B., Saeedi F., Talebi M., Shakibaei M., Samarghandian S. (2021). Impact of Metformin on Cancer Biomarkers in Non-Diabetic Cancer Patients: A Systematic Review and Meta-Analysis of Clinical Trials. Curr. Oncol..

[134] Goodwin P.J., Dowling R.J.O., Ennis M., Chen B.E., Parulekar W.R., Shepherd L.E., Burnell M.J., Vander Meer R., Molckovsky A., Gurjal A. (2021). Effect of metformin versus placebo on metabolic factors in the MA.32 randomized breast cancer trial. NPJ Breast Cancer.

[135] Pimentel I., Chen B.E., Lohmann A.E., Ennis M., Ligibel J., Shepherd L., Hershman D.L., Whelan T., Stambolic V., Mayer I. (2021). The Effect of Metformin vs Placebo on Sex Hormones in Canadian Cancer Trials Group MA.32. JNCI J. Natl. Cancer Inst..

[136] Liu F., Wang C., Zhang L., Xu Y., Jang L., Gu Y., Cao X., Zhao X., Ye J., Li Q. (2014). Metformin prevents hepatic steatosis by regulating the expression of adipose differentiation-related protein. Int. J. Mol. Med..

[137] Lin H.Z., Yang S.Q., Chuckaree C., Kuhajda F., Ronnet G., Diehl A.M. (2000). Metformin reverses fatty liver disease in obese, leptin-deficient mice. Nat. Med..

[138] Kita Y., Takamura T., Misu H., Ota T., Kurita S., Takeshita Y., Uno M., Matsuzawa-Nagata N., Kato K.-I., Ando H. (2012). Metformin Prevents and Reverses Inflammation in a Non-Diabetic Mouse Model of Nonalcoholic Steatohepatitis. PLoS ONE.

[139] Marchesini G., Brizi M., Bianchi G., Tomassetti S., Zoli M., Melchionda N. (2001). Metformin in non-alcoholic steatohepatitis. Lancet.

[140] Loomba R., Lutchman G., Kleiner D.E., Ricks M., Feld J.J., Borg B.B., Modi A., Nagabhyru P., Sumner A.E., Liang T.J. (2009). Clinical trial: Pilot study of metformin for the treatment of non-alcoholic steatohepatitis. Aliment. Pharmacol. Ther..

[141] Uygun A., Kadayifci A., Isik A.T., Ozgurtas T., Deveci S., Tuzun A., Yesilova Z., Gulsen M., Dagalp K. (2004). Metformin in the treatment of patients with non-alcoholic steatohepatitis. Aliment. Pharmacol. Ther..

[142] Bugianesi E., Gentilcore E., Manini R., Natale S., Vanni E., Villanova N., David E., Rizzetto M., Marchesini G. (2005). A randomized controlled trial of metformin versus vitamin E or prescriptive diet in nonalcoholic fatty liver disease. Am. J. Gastroenterol..

[143] Omer Z., Cetinkalp S., Akyildiz M., Yilmaz F., Batur Y., Yilmaz C., Akarca U. (2010). Efficacy of insulin-sensitizing agents in nonalcoholic fatty liver disease. Eur. J. Gastroenterol. Hepatol..

[144] Haukeland J.W., Konopski Z., Eggesbø H.B., Von Volkmann H.L., Raschpichler G., Bjøro K., Haaland T., Løberg E.M., Birkeland K. (2009). Metformin in patients with non-alcoholic fatty liver disease: A randomized, controlled trial. Scand. J. Gastroenterol..

[145] Lavine J.E., Schwimmer J.B., Van Natta M.L., Molleston J.P., Murray K.F., Rosenthal P., Abrams S.H., Scheimann A.O., Sanyal A.J., Chalasani N. (2011). Effect of vitamin E or metformin for treatment of nonalcoholic fatty liver disease in children and adolescents: The TONIC randomized controlled trial. JAMA.

[146] Krakoff J., Clark J.M., Crandall J.P., Wilson C., Molitch M.E., Brancati F.L., Edelstein S.L., Knowler W.C. (2010). Effects of metformin and weight loss on serum alanine aminotransferase activity in the diabetes prevention program. Obesity.

[147] Li Y., Liu L., Wang B., Wang J., Chen D. (2013). Metformin in non-alcoholic fatty liver disease: A systematic review and meta-analysis. Biomed. Rep..

[148] Ou Z., Kong X., Sun X., He X., Zhang L., Gong Z., Huang J., Xu B., Long D., Li J. (2018). Metformin treatment prevents amyloid plaque deposition and memory impairment in APP/PS1 mice. Brain Behav. Immun..

[149] Hardie D.G. (2014). AMP-Activated Protein Kinase: Maintaining Energy Homeostasis at the Cellular and Whole-Body Levels. Annu. Rev. Nutr..

[150] Jin Q., Cheng J., Liu Y., Wu J., Wang X., Wei S., Zhou X., Qin Z., Jia J., Zhen X. (2014). Improvement of functional recovery by chronic metformin treatment is associated with enhanced alternative activation of microglia/macrophages and increased angiogenesis and neurogenesis following experimental stroke. Brain Behav. Immun..

[151] Hohnholt M.C., Blumrich E.-M., Waagepetersen H.S., Dringen R. (2017). The antidiabetic drug metformin decreases mitochondrial respiration and tricarboxylic acid cycle activity in cultured primary rat astrocytes. J. Neurosci. Res..

[152] Mostafa D.K., Ismail C.A., Ghareeb D.A. (2016). Differential metformin dose-dependent effects on cognition in rats: Role of Akt. Psychopharmacology.

[153] McNeilly A.D., Williamson R., Balfour D.J.K., Stewart C.A., Sutherland C. (2012). A high-fat-diet-induced cognitive deficit in rats that is not prevented by improving insulin sensitivity with metformin. Diabetologia.

[154] Oliveira W.H., Nunes A.K., França M.E.R., Santos L.A., Lós D.B., Rocha S.W., Barbosa K.P., Rodrigues G.B., Peixoto C.A. (2016). Effects of metformin on inflammation and short-term memory in streptozotocin-induced diabetic mice. Brain Res..

[155] Ghadernezhad N., Khalaj L., Pazoki-Toroudi H., Mirmasoumi M., Ashabi G. (2016). Metformin pretreatment enhanced learning and memory in cerebral forebrain ischaemia: The role of the AMPK/BDNF/P70SK signalling pathway. Pharm. Biol..

[156] Chaudhari K., Reynolds C.D., Yang S.-H. (2020). Metformin and cognition from the perspectives of sex, age, and disease. GeroScience.

[157] Moore E., Mander A., Ames D., Carne R., Sanders K., Watters D. (2012). Cognitive impairment and vitamin B12: A review. Int. Psychogeriatr..

[158] Moore E.M., Mander A.G., Ames D., Kotowicz M.A., Carne R.P., Brodaty H., Woodward M., Boundy K., Ellis K.A., Bush A.I. (2013). Increased Risk of Cognitive Impairment in Patients With Diabetes Is Associated With Metformin. Diabetes Care.

[159] Porter K.M., Ward M., Hughes C.F., O’Kane M., Hoey L., McCann A., Molloy A.M., Cunningham C., Casey M.C., Tracey F. (2019). Hyperglycemia and Metformin Use Are Associated With B Vitamin Deficiency and Cognitive Dysfunction in Older Adults. J. Clin. Endocrinol. Metab..

[160] Ng T.P., Feng L., Yap K.B., Lee T.S., Tan C.H., Winblad B. (2014). Long-term metformin usage and cognitive function among older adults with diabetes. J. Alzheimer’s Dis..

[161] Samaras K., Makkar S., Crawford J.D., Kochan N.A., Wen W., Draper B., Trollor J.N., Brodaty H., Sachdev P.S. (2020). Metformin Use Is Associated With Slowed Cognitive Decline and Reduced Incident Dementia in Older Adults With Type 2 Diabetes: The Sydney Memory and Ageing Study. Diabetes Care.

[162] Koenig A.M., Mechanic-Hamilton D., Xie S.X., Combs M.F., Cappola A.R., Xie L., Detre J.A., Wolk D.A., Arnold S.E. (2017). Effects of the Insulin Sensitizer Metformin in Alzheimer Disease. Alzheimer Dis. Assoc. Disord..

[163] Guo M., Mi J., Jiang Q.M., Xu J.M., Tang Y.Y., Tian G., Wang B. (2014). Metformin may produce antidepressant effects through improvement of cognitive function among depressed patients with diabetes mellitus. Clin. Exp. Pharmacol. Physiol..

[164] Luchsinger J.A., Ma Y., Christophi C.A., Florez H., Golden S.H., Hazuda H., Crandall J., Venditti E., Watson K., Jeffries S. (2017). Metformin, Lifestyle Intervention, and Cognition in the Diabetes Prevention Program Outcomes Study. Diabetes Care.

[165] Campbell J.M., Stephenson M.D., de Courten B., Chapman I., Bellman S.M., Aromataris E. (2018). Metformin Use Associated with Reduced Risk of Dementia in Patients with Diabetes: A Systematic Review and Meta-Analysis. J. Alzheimer’s Dis..

